# An HIV Vaccine Protective Allele in *FCGR2C* Associates With Increased Odds of Perinatal HIV Acquisition

**DOI:** 10.3389/fimmu.2021.760571

**Published:** 2021-11-30

**Authors:** Joy Ebonwu, Ria Lassaunière, Maria Paximadis, Mark Goosen, Renate Strehlau, Glenda E. Gray, Louise Kuhn, Caroline T. Tiemessen

**Affiliations:** ^1^ Division of Public Health Surveillance and Response, National Institute for Communicable Diseases, Johannesburg, South Africa; ^2^ Faculty of Health Sciences, University of the Witwatersrand, Johannesburg, South Africa; ^3^ Virus Research and Development Laboratory, Department of Virus and Microbiological Special Diagnostics, Statens Serum Institut, Copenhagen, Denmark; ^4^ Centre for HIV & STIs, National Institute for Communicable Diseases, Johannesburg, South Africa; ^5^ Empilweni Services and Research Unit, Rahima Moosa Mother and Child Hospital, Johannesburg, South Africa; ^6^ Department of Paediatrics and Child Health, Faculty of Health Sciences, University of the Witwatersrand, Johannesburg, South Africa; ^7^ Perinatal HIV Research Unit, Faculty of Health Sciences, University of the Witwatersrand, Johannesburg, South Africa; ^8^ South African Medical Research Council, Cape Town, South Africa; ^9^ Gertrude H. Sergievsky Centre, College of Physicians and Surgeons, Columbia University, New York, NY, United States; ^10^ Department of Epidemiology, Mailman School of Public Health, Columbia University, New York, NY, United States

**Keywords:** Fc gamma receptor, FCGR2C, genetic variant, polymorphism, gene copy number, perinatal HIV-1 acquisition, genetic association study, South Africa

## Abstract

In the Thai RV144 HIV-1 vaccine trial, a three-variant haplotype within the Fc gamma receptor 2C gene (*FCGR2C*) reduced the risk of HIV-1 acquisition. A follow-on trial, HVTN702, of a similar vaccine candidate found no efficacy in South Africa, where the predominant population is polymorphic for only a single variant in the haplotype, c.134-96C>T (rs114945036). To investigate a role for this variant in HIV-1 acquisition in South Africans, we used the model of maternal-infant HIV-1 transmission. A nested case-control study was conducted of infants born to mothers living with HIV-1, comparing children with perinatally-acquired HIV-1 (cases, n = 176) to HIV-1-exposed uninfected children (controls, n = 349). All had received nevirapine for prevention of mother-to-child transmission. The *FCGR2C* copy number and expression variants (c.−386G>C, c.−120A>T c.169T>C, and c.798+1A>G) were determined using a multiplex ligation-dependent probe amplification assay and the c.134-96C>T genotype with Sanger sequencing. The copy number, genotype and allele carriage were compared between groups using univariate and multivariate logistic regression. The *FCGR2C* c.134-96C>T genotype distribution and copy number differed significantly between HIV-1 cases and exposed-uninfected controls (*P* = 0.002, *P*_Bonf_ = 0.032 and *P* = 0.010, *P*_Bonf_ = > 0.05, respectively). The *FCGR2C* c.134-96T allele was overrepresented in the cases compared to the controls (58% *vs* 42%; *P* = 0.001, *P*_Bonf_ = 0.016). Adjusting for birthweight and *FCGR2C* copy number, perinatal HIV-1 acquisition was associated with the c.134-96C>T (AOR = 1.89; 95% CI 1.25-2.87; *P* = 0.003, *P*_Bonf_ = 0.048) and c.169C>T (AOR = 2.39; 95% CI 1.45-3.95; *P* = 0.001, *P*_Bonf_ = 0.016) minor alleles but not the promoter variant at position c.−386G>C. The c.134-96C>T variant was in strong linkage disequilibrium with the c.169C>T variant, but remained significantly associated with perinatal acquisition when adjusted for c.169C>T in multivariate analysis. In contrast to the protective effect observed in the Thai RV144 trial, we found the *FCGR2C* variant c.134-96T-allele associated with increased odds of perinatal HIV-1 acquisition in South African children. These findings, taken together with a similar deleterious association found with HIV-1 disease progression in South African adults, highlight the importance of elucidating the functional relevance of this variant in different populations and vaccination/disease contexts.

## Introduction

The crystallisable fragment (Fc) region of immunoglobulin G (IgG) antibodies interacts with Fc gamma receptors (FcγRs) expressed on the surface of hematopoietic cells to mediate effector functions. In humans, FcγRs are divided into three classes (FcγRI, FcγRII, and FcγRIII) based on structural domain organization, differences in affinity and specificity for IgG subclasses, and whether their binding triggers activating or inhibitory signals. The low affinity FcγRs are encoded by five genes on chromosome 1q23, namely *FCGR2A, FCGR2B, FCGR2C*, *FCGR3A* and *FCGR3B* (1) and play different roles in regulating immune responses (2). Functionally significant genetic variants occur for all low affinity FcγRs. These affect FcγRs by altering receptor cell surface density, binding affinities to IgGs, glycosylation patterns, cellular distribution, or subcellular localization (3, 4). Apart from single nucleotide polymorphisms (SNPs), copy number variation (CNV) has been demonstrated for *FCGR2C*, *FCGR3A and FCGR3B* (5, 6), and has been correlated with protein expression levels (7). Genes are duplicated or deleted at the *FCGR2/3* locus within well-defined copy number variable regions (CNRs), namely CNR1, CNR2, CNR3 (8, 9) and CNR4 (9). The most common are CNR1, which comprises genes of *FCGR2C*, *HSPA7* and *FCGR3B* and CNR2 that includes the distal part of *FCGR2A* (exon 8 and 3’-untranslated region [3’UTR]), *HSPA6, FCGR3A* and proximal part of *FCGR2C* (excluding exon 8 and 3’UTR) (9).

The *FCGR2C* gene, encoding FcγRIIc, is described as a pseudogene and is the product of an unequal crossover event between the 5’ part of *FCGR2B* genes and 3’ part of *FCGR2A* (10). Expression of the membrane-bound FcγRIIc protein depends on a combination of three minor alleles that include the c.169T>C variant in exon 3, which substitutes a premature stop codon with a glutamine at amino acid 57, and two splice variants in intron 7 - c.798+1A>G and c.799-1G>C (6, 11). Due to significant variation of the minor allele frequencies in different populations (12), FcγRIIc protein expression is subject to ethnic variation. The splice variant c.798+1A>G minor allele rarely occurs in black Africans and East Asians, thus, few individuals in this population express FcγRIIc compared to approximately 33% of Caucasians (12). An additional *FCGR2C* c.134-96C>T variant (also known as *FCGR2C* 126C>T) has been identified as clinically significant (13). Overall, genetic variation of *FCGR2C* has been associated with rheumatoid arthritis (14) idiopathic thrombocytopenic purpura (15), HIV-tuberculosis co-infection (16), antibody responses to vaccinations (11, 13, 17) and HIV disease progression (18).

In the RV144 vaccine trial, where the vaccine regimen was designed against HIV-1 clade B and E, a three-variant haplotype within *FCGR2C* [c.353C>T (rs138747765); c.391+111G>A (rs78603008) and c.134-96C>T (rs114945036)] reduced the risk of HIV-1 acquisition in Thai adults. The vaccine test subjects carrying at least one minor allele of the c.134-96C>T tag variant had an estimated vaccine efficacy of 91% against the CRF01_AE 169K HIV-1 strain and 64% against any HIV-1 strain, while those with wild type allele exhibited a vaccine efficacy of 15% and 11%, respectively (13). Conversely, two variants within the haplotype were associated with increased risk of HIV-1 acquisition in the HIV Vaccine Trials Network (HVTN) 505 vaccine trial (17). A follow-on trial of a similar vaccine regime to RV144 (HVTN 702 vaccine trial) tested in South Africa showed no efficacy (19). The cause underlying the different vaccine trial outcomes remains undetermined. However, differences in vaccine regimen, population, demographics and environment should be considered (17). A role for population genetics warrants consideration, since black South Africans do not possess the complete Thai *FCGR2C* haplotype and are only polymorphic for c.134-96C>T (rs114945036) (12).

The c.134-96C>T *FCGR2C* variant has been implicated in HIV-1 disease progression in a black South African cohort (18). However, unlike the protective effect observed for Thai vaccinees, the minor allele was associated with increased odds of HIV-1 disease progression in those already infected. It is unknown whether the alternate protective and deleterious roles of the *FCGR2C* c.134-96C>T variant in the Thai vaccinees and HIV-1 infected South Africans is due to different mechanisms involved before and after HIV-1 infection or whether the genetic differences associated with the haplotype alters its role in the two populations. Establishing the role of the c.134-96C>T variant in HIV-1 protective immunity in other models of persistent HIV-1 exposure, such as infants born to HIV-1 infected mothers, will be informative.

Mother-to-child transmission (MTCT) is an attractive model in which to study immune correlates of protection since both members of the transmitting dyad are known, timing of transmission can be ascertained with reasonable precision, and it affords the opportunity to assess factors contributing to both the infectiousness of the transmitter (mother) and susceptibility of the recipient (infant) (20, 21). Limitations of this model are that transmission occurs between genetically similar individuals, exposure to HIV-1 occurs at a time of early immune development, and immune circumstances during pregnancy are associated with tolerance of the fetal allograft (22). Nevertheless, it provides a unique opportunity to investigate the role of FcγR-mediated effector functions, since the individual (fetus/infant) at risk is passively immunized with HIV-1-specific antibodies through trans-placental transfer of IgG from the HIV-1 infected mother and the model is not confounded by interspecies differences as observed for non-human primate studies (23). In this study, we investigate the association between the *FCGR2C* c.134-96C>T variant and HIV-1 acquisition in black South African children born to women living with HIV.

## Materials and Methods

### Study Design and Population

A nested case-control study was undertaken to investigate the association between the *FCGR2C* variants and HIV-1 perinatal acquisition in children, combining data from past studies of five perinatal cohorts at two hospitals in Johannesburg, South Africa (24–27). One of the five cohorts consists of 546 HIV-infected children who were recruited as part of two sequential randomized clinical trials (NEVEREST 2 and 3) (24–26). The remaining four cohorts comprised of 849 HIV-1 infected mothers and their infants who were recruited and followed prospectively, of whom 83 (10%) infants acquired HIV (27). In the present study, only samples that were found and with sufficient volume were genotyped. *FCGR2C* genotypic data from 99 out of 546 and 77 of 83 HIV-1-infected children (cases) from the NEVEREST and mother-infant cohorts, respectively (n = 176) were compared with 349 of the HIV-exposed uninfected children (controls).

Mode of transmission was defined according to the presence or absence of detectable HIV-1 deoxyribonucleic acid (DNA) in the infant at birth and six weeks of age. Infants that tested HIV-1 positive at six weeks of age, but who were negative at birth, were considered to be infected intrapartum (during labor and delivery) (n = 31), while infants that tested HIV-1 positive at birth were considered infected *in utero* (n = 19). Infants who were HIV-1 positive at six weeks, but had no birth sample, were categorized as ‘undetermined’ (n = 28). In the ‘undetermined’ category, 25/28 (89.2%) mothers received single-dose nevirapine or triple-drug combination therapy (two nucleoside reverse transcriptase inhibitors with either a protease inhibitor or non-nucleoside reverse transcriptase inhibitor) known to reduce intrapartum transmission (27–29). Genotyping generated a result for all the *FCGR2C* variants assessed in this study in 27 out of the 28 samples. It was thus concluded that the majority (n = 27) of infants were likely infected *in utero* and were combined with the *in utero* group to form an *in utero*-enriched group. For the NEVEREST cohort, there were no birth samples as the children were recruited from six weeks of life. They were therefore classified as mixed transmission since a few were breastfeeding infections and *in utero* infections could not be distinguished from intrapartum infections (n = 99). All study participants were black South Africans and received nevirapine for prevention of MTCT. Maternal antiretroviral therapy was not routinely used at the time.

### Ethics

Ethics approval for the study was obtained from the University of the Witwatersrand Human Research Ethics Committee (Reference numbers: M170585; M180575).

### Genotyping

*FCGR2C* copy number and SNPs that affect gene expression – c.169T>C (p.X57Q), c.798+1A>G, and the *FCGR2B/C* promoter variant at position c.−386G>C and c.−120A>T – were determined using the *FCGR*-specific multiplex ligation-dependent probe amplification assay (MRC Holland, Amsterdam, The Netherlands) according to manufacturer’s instructions. Amplicons were separated by capillary electrophoresis on an ABI Genetic Analyser 3130 (Life Technologies, Applied Bio systems, Foster City, CA, USA) and fragments analyzed with the Coffalyzer.NET software (MRC Holland) using peak height as a measure of gene/allele copy number. We did not utilize gene-specific polymerase chain reactions (PCR) to distinguish *FCGR2B* and *FCGR2C* promoter sequences since earlier findings indicate that African individuals do not possess the promoter variant in *FCGR2B*, and thus any detected c.−386G>C minor alleles were in *FCGR2C* (12).

The *FCGR2C* c.134-96C>T (rs114945036) variant was genotyped through conventional PCR and Sanger nucleotide sequencing. In brief, a 6,374 base pair fragment was amplified with the Expand Long Template PCR System (Roche, Mannheim, Germany) using the *FCGR2B/C* sense primer (5’-ATGTATGGGGTGTCTGTGTGTC-3’) and *FCGR2C*-specific antisense primer (5’-CTCAAATTGGGCAGCCTTCAC-3’) (15). The PCR reaction consisted of ~20 ng genomic DNA as template, 3.75 U Expand Long Template enzyme mix, 5 µl 10× PCR buffer 3 (2.75 mM MgCl_2_), 500 µM of each deoxynucleotide, 0.3 µM of each oligonucleotide primer, and molecular grade water to a final volume of 50 µl. The PCR conditions were initial denaturation at 94°C for 2 minutes, followed by 10 cycles of 94°C for 10 seconds (denaturation), 60°C for 15 seconds (annealing) and 68°C for 7 minutes (elongation). Thereafter, 25 cycles repeat process of denaturation, annealing and elongation respectively at 94°C for 15 seconds, 60°C for 15 seconds and 68°C for 7 minutes plus 20 seconds cycle elongation for each successive cycle; and a final elongation cycle at 72°C for 7 minutes. The internal antisense primer (5’-CCTCCACTGACCAGAAAGCAC-3’) was used in standard BigDye Terminator v3.1 Cycle Sequencing reactions. Sequences were analyzed in Sequencer version 4.5 (Gene Codes Corporation, Ann Arbor, MI) and area under the curve of the electropherogram used to determine allele count for individuals bearing more than two *FCGR2C* gene copies.

### Nomenclature

The SNP nomenclature used in this manuscript refers to the amino acid positions in the full protein, in accordance with the Human Genome Variation Society (HGVS) guidelines (30). The numbering of nucleotides is according to the Genome Reference Consortium Human Reference 38 [GRCh38 (hg38)].

### Statistical Analysis

Categorical data were summarized as proportions and the Fisher’s Exact test was used for comparisons. For numerical data, the t-test was used for comparison of means. Univariate and multivariate analyses were conducted to determine factors associated with perinatal HIV acquisition. Adjustment for multiple comparisons was performed using the Bonferroni correction, which considered 16 independent tests — four unrelated clinical subgroups each tested for four variants (gene copy number, c.134-96C>T, c.169T<C, and c.−386G>C). Both unadjusted and adjusted P values are reported. Analysis of an association between *FCGR2C* variants and HIV-1 acquisition was limited to variants whose allele frequencies were ≥ 5%. Due to the low frequencies of minor allele homozygotes, their effect was tested using dominant model approach, where participants were divided into two genotype groups: homozygous genotype of the major allele and the two genotypes containing at least one minor allele. All analyses were performed in STATA version 15.1 (StataCorp LP, Texas, USA).

Linkage disequilibrium between *FCGR2C* functional variants and CNRs was computed using the Haploview software package (31) and expressed as D prime (D′) and square of the correlation coefficient (r^2^). The closer D′ is to 1 the stronger the LD between two loci. Hardy-Weinberg equilibrium was considered for individuals with two gene copies and the statistics abstracted from the Haploview analysis output. For the analysis, genotypic data with multiple gene copies were considered homozygous if all copies carried the same allele or heterozygous when both alleles were present.

## Results

### Cohort

This nested case-control study investigated *FCGR2C* genotypic data from 525 children to determine the role of *FCGR2C* variants and HIV-1 acquisition in South African children born to women living with HIV-1. The cohort includes 176 HIV-1 infected (cases) and 349 HIV-exposed-uninfected (controls) children. The HIV-1 infected children comprised four transmission mode groups: *in utero* (n = 19), *in utero*-enriched (n = 46), intrapartum (n = 31) and mixed (n = 99). Overall, there was no significant difference in sex, gestation and breastfeeding status between the HIV-1 infected and HIV-1 uninfected cohort. However, the total HIV-1 infected and HIV-1 exposed-uninfected groups differed significantly in birth weight at delivery. Specifically, a higher proportion of HIV-infected children had a birth weight below 2500 g (22% *vs.* 11%; *P* = 0.001, *P*_Bonf_ = 0.016) (**Table 1**).

**Table 1:** Demographic and clinical characteristics of perinatal HIV-1 acquisition groups.

Variables	HIV-1-exposed uninfected	Total HIV-1 infected		*In utero* infected		*In utero*-enriched infected		Mixed infected		Intrapartum infected	
	n = 349	n = 176	*P* value	n = 19	*P* value	n = 46	*P* value	n = 99	*P* value	n = 31	*P* value
Sex	(n = 346)	(n = 176)	1.000		0.487		0.875		0.569		0. 260
Male	160 (46)	80 (45)		7 (37)		20 (43)		42 (42)		18 (58)	
Female	189 (54)	96 (55)		12 (63)		26 (57)		57 (58)		13 (42)	
Gestation	(n = 327)	(n = 164)	0.355	(n = 18)	0.105	(n = 44)	0.788	(n = 95)	1.000	(n = 25)	**0.013** (*P*_Bonf_ = 0.208)
Term	295 (90)	143 (87)		14 (78)		39 (89)		86 (91)		18 (72)	
Preterm (<37 weeks)	32 (10)	21 (13)		4 (22)		5 (11)		9 (9)		7 (28)	
Birth weight (g)	(n = 344)	(n = 168)	**0.001** **(*P*_Bonf_ =** **0.016)**		0.251		**0.003** **(*P*_Bonf_ =** **0.048)**	(n = 92)	**0.001** **(*P*_Bonf_ =** **0.016)**	(n = 30)	0.898
≥2500	307 (89)	131 (78)		15 (79)		34 (74)		70 (76)		27 (90)	
<2500	37 (11)	37 (22)		4 (21)		12 (26)		22 (24)		3 (10)	
Breastfed	(n = 346)	(n = 170)	1.000	(n = 18)	0.384	(n = 45)	0.117	(n = 95)	0.780	(n = 30)	0.359
No	271 (78)	134 (79)		16 (89)		40 (89)		73 (77)		21 (70)	
Yes	75 (22)	36 (21)		2 (11)		5 (11)		22 (23)		9 (30)	

Data are n (%) unless otherwise specified.

The P values refer to comparisons between the HIV-1-exposed uninfected (control) group and each of the HIV-1 infected (case) groups.

Bold indicates statistical significance of P < 0.05; P_Bonf_, Bonferroni corrected P value.

### *FCGR2C* Copy Number Distribution and HIV-1 Acquisition

**Figure 1:**
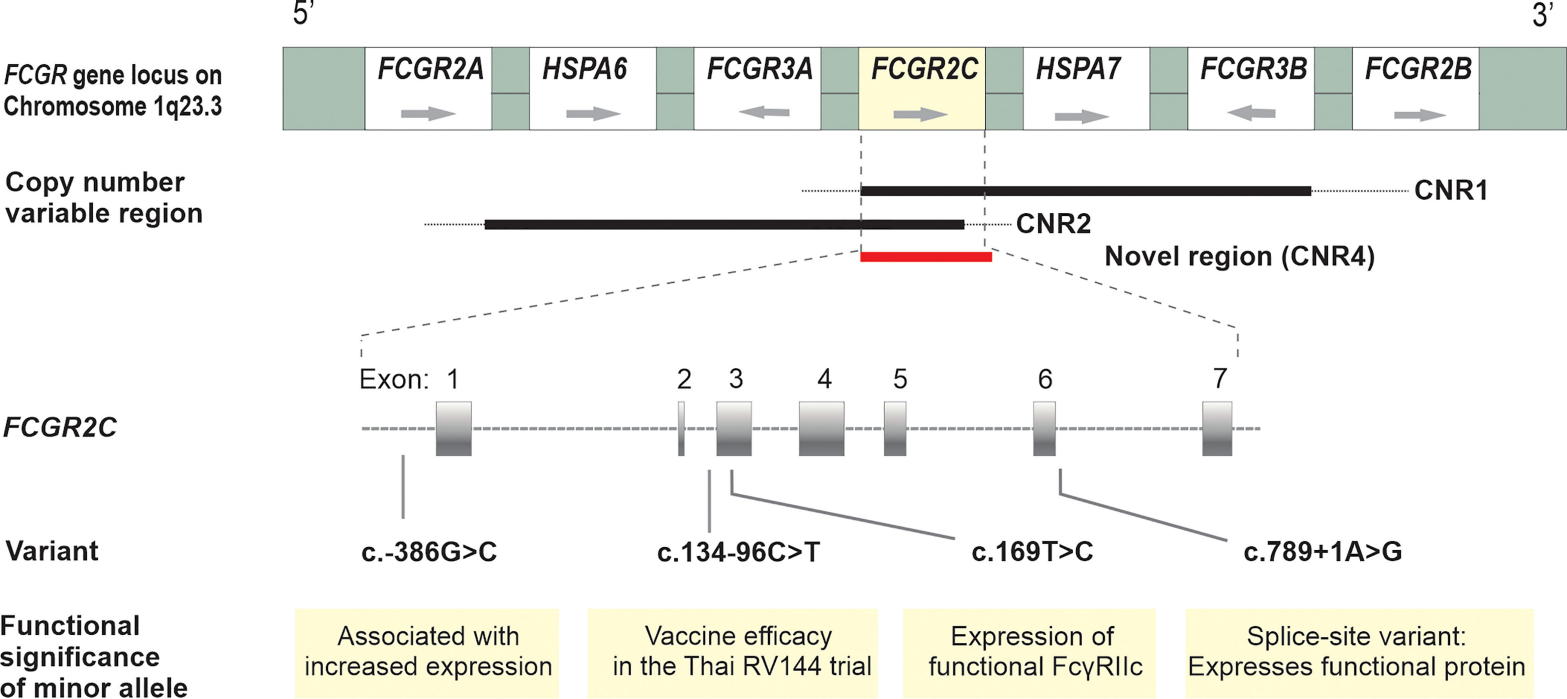
Schematic diagram of the *FCGR* gene locus on chromosome 1q23 with their orientation; CNV of *FCGR2C* within distinct copy number variable regions – previously designated CNR1, CNR2 (8,^9^) and a novel region (CNR4) where only *FCGR2C* duplicated/deleted, as described among South African black population (12); Functional and clinically significant *FCGR2C* variants and their positions on the gene.

The *FCGR2C* gene, highly homologous to *FCGR2B* in the first six exons and *FCGR2A* in the last two exons, is subject to CNV within previously described distinct regions (**Figure 1**). We did not observe any individual with a complete absence of the *FCGR2C* gene. Overall, *FCGR2C* CNV occurred in 166/525 (32%) children, with the frequency of duplications (n = 114) 2.2-fold higher than deletions (n = 52) (69% *vs.* 31%). The copy number distribution was significantly different between the HIV-1 infected and HIV-1 exposed-uninfected groups but not after Bonferroni correction (*P* = 0.010, *P*_Bonf_ > 0.05) (**Table 2**). Variation in copy number among the whole study cohort was observed more frequently in CNR1, which encompasses a complete *FCGR2C* copy, *HSPA7* and *FCGR3B* (28%; n = 147) than CNR2, with an incomplete *FCGR2C* copy, *HSPA6* and *FCGR3A* (2.7%; n = 14). In six instances (1.1%), we observed CNV for only *FCGR2C* in the absence of duplicated/deleted flanking genes, as previously described among the South African black population (12). A duplication in both CNR1 and CNR2 was observed in one individual. Given the differences between the CNRs, their copy number variability was determined separately.

**Table 2:** *FCGR2C* copy number distribution in HIV-1-exposed infected and uninfected infants.

Variables	Total study cohort	HIV-1-exposed uninfected	TotalHIV-1 infected		*In utero* infected		*In utero*-enriched infected		Mixed infected		Intrapartum infected	
	n = 525	n = 349	n = 176	*P* value	n = 19	*P* value	n = 46	*P* value	n = 99	*P* value	n = 31	*P* value
*FcγRIIc* copy number				**0.010** (*P*_Bonf_ = 0.16**)**		0.672		0.142		0.095		**0.028** (*P*_Bonf_ = 0.448)
1 copy	52 (10)	44 (12)	8 (4)		1 (5.3)		3 (6)		5 (5)		0 (0)	
2 copies	359 (68)	233 (67)	126 (72)		13 (68.4)		28 (61)		71 (72)		27 (87)	
≥3 copies	114 (22)	72 (21)	42 (24)		5 (26.3)		15 (33)		23 (23)		4 (13)	
CNR1	(n = 147)	(n = 105)	(n = 42)	**0.009** (*P*_Bonf_ = 0.144)	(n = 5)	0.162	(n = 15)	**0.035** (*P*_Bonf_ = 0.56)	(n = 23)	0.228	(n = 4)	0.296
deletion	44 (30)	38 (36)	6 (14)		0 (0)		1 (7)		5 (22)		0 (0)	
duplication	103 (70)	67 (64)	36 (86)		5 (100)		14 (93)		18 (78)		4 (100)	
CNR2	(n = 14)	(n = 8)	(n = 6)	0.767	(n = 1)	0.444	(n = 2)	0.444	(n = 4)	0.180	(n = 0)	
deletion	5 (36)	3 (37.5)	2 (33)		1(100)		2 (100)		0		0	
duplication	9 (64)	5 (62.5)	4 (67)		0 (0)		0		4 (100)		0	
CNR4	(n = 6)	(n = 3)	(n = 3)	0.100	(n = 0)		(n = 1)	0.250	(n = 2)	0.100	(n = 0)	
deletion	3 (50)	3 (100)	0		0		0		0)		0	
duplication	3 (50)	0	3 (100)		0		1 (100)		2(100)		0	

Data are n (%) unless otherwise specified.

The P values refer to comparisons between the HIV-1-exposed uninfected (control) group and each of the HIV-1 infected (case) groups.

Bold indicates statistical significance of P < 0.05; P_Bonf_, Bonferroni corrected P value.

Within CNR1, CNV was significantly different between the HIV-1 infected and HIV-1 exposed-uninfected groups (*P* = 0.009, *P*_Bonf_ > 0.05). This difference was primarily determined by gene deletions. There were a higher number of HIV-1 exposed-uninfected children with a single gene copy compared to HIV-1 infected children (36% *vs.* 14%) (**Table 2**). Using two *FCGR2C* gene copies as reference, the possession of a single gene copy was independently associated with reduced odds of HIV-1 acquisition (OR = 0.29; 95% CI 0.12-0.71; *P* = 0.007, *P*_Bonf_ > 0.05) and retained significance after controlling for birthweight and *FCGR2C* genotypes (AOR = 0.37; 95% CI 0.15-0.90; *P* = 0.029, *P*_Bonf_ > 0.05) (**Table 3**). The CNR2 and the novel CNR4 variability were excluded from further association analysis due to low frequencies (< 5%).

**Table 3:** Effect of *FCGR2C* CNR1 copy number distribution on perinatal HIV-1 acquisition, adjusting for birthweight and *FCGR2C* genotypes.

*FcγRIIc* copy number (Total group)	Univariate		Multivariate	
	OR (95% CI)	P-value	Adjusted OR	P-value
1 copy	0.29 (0.12-0.71)	**0.007** (*P*_Bonf_ = 0.112)	0.37 (0.15-0.90)	**0.029** (*P*_Bonf_ = 0.464)
2 copies	Ref		Ref	
≥3 copies	0.99 (0.63-1.57)	0.978	0.74 (0.43-1.27)	0.275

OR, Odds Ratio; CI, Confidence Interval; P_Bonf_, Bonferroni corrected P value.

Bold indicates statistical significance of P < 0.05.

### *FCGR2C* Variants That Determine the Expression of Surface FcγRIIc

We further genotyped functional *FCGR2C* variants that determine the expression of FcγRIIc (c.−386G>C, c.169T>C and c.798+1A>G) and the c.134-96C>T variant that associated with risk of HIV-1 acquisition in the Thai RV144 HIV-1 vaccine trial (**Figure 1**). To assess the role of the *FCGR2C* genotypes and allele distribution in perinatal HIV-1 acquisition, children with a single *FCGR2C* gene copy were considered homozygous; those with more than one *FCGR2C* gene copy were considered homozygous if all the alleles were the same or heterozygous if both alleles were present. With MLPA, we obtained genotypic data from 166 out of the 176 HIV-1 infected for c.169T>C and c.−386G>C variants.

A SNP in exon 3 (c.169T>C) that results in an open reading frame (ORF) or a stop codon determines the expression of FcγRIIc when present with the minor allele of two splice variants in intron 7 (c.798+1A>G and c.799-1G>C). While 129/515 (25%) of individuals carried at least one *FCGR2C-*ORF (c.169C allele), only 4/129 (3%) individuals possessed the c.798+1G minor allele that represents the classic *FCGR2C*-ORF and predicts FcγRIIc expression (6, 32). Of the four individuals with the c.798+1G minor allele, three were HIV-1-exposed uninfected and one HIV-1 infected. Conversely, the c.169C allele co-occurred with the c.798+1A allele in 97% (n = 125) of the participants, representing the non-classic *FCGR2C*-ORF that does not yield surface expression of FcγRIIc. The c.799-1G>C splice cite variant was not genotyped, since it has been shown that the c.169C and c.798+1A alleles are syntenic with the c.799-1G allele in South African population (12).

**Figure 2:**
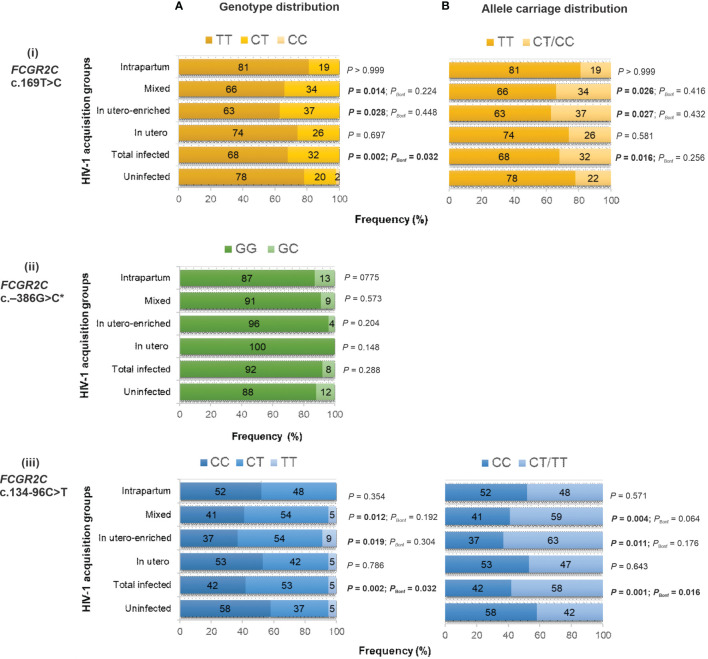
Genotype **(A)** and allele carriage **(B)** distribution of functional *FCGR2C* variants and their association with perinatal HIV-1 acquisition in black South Africans [HIV-1-exposed uninfected (n = 349), total infected (n = 176 for c.134-96C>T; n = 166 for c.169T>C and c.−386G>C), *in utero* infected (n = 19), *in utero*-enriched infected (n = 46), mixed infected (n = 99 for c.134-96C>T; n = 89 for c.169T>C and c.−386G>C) and intrapartum infected (n = 31)]. * No individual with homozygous −386 CC genotype.

While the c.169T>C variant alone does not result in surface expression of FcγRIIc, it may have other unknown functional consequences and was therefore investigated for a possible association with HIV-1 perinatal transmission. The c.169T>C genotype distribution was significantly different between HIV-1 infected and HIV-1 exposed-uninfected children (*P* = 0.002, *P*_Bonf_ = 0.032) (**Figure 2**i). In a dominant model, the c.169C was overrepresented in the HIV-1 infected compared to the uninfected children (32% *vs.* 22%) and significantly associated with increased odds of HIV acquisition (OR = 1.68; 95% CI 1.11-2.55; *P* = 0.013, *P*_Bonf_ > 0.05). The strength of association increased after adjusting for birthweight and CNR1 copy number (AOR = 2.39; 95% CI 1.45-3.95; *P* = 0.001, *P*_Bonf_ = 0.016). For the *FCGR2B/C* promoter variant at position –386G>C, which modulates gene expression levels, no significant difference in genotype frequency was observed between the HIV-1 infected and HIV-1 uninfected cohort (*P* = 0.288) (**Figure 2**ii). The homozygous −386 CC genotype was not observed in any individual. Due to the low frequency of the splice site variant c.798+1A>G minor allele, an association analysis was not conducted.

### The Thai *FCGR2C* Haplotype Tag Variant c.134-96C>T Associates With Increased Odds of Perinatal HIV-1 Transmission

The *FCGR2C* c.134-96C>T genotype distribution was significantly different between the children who acquired HIV-1 and those who did not (*P* = 0.002, *P*_Bonf_ = 0.032) (**Figure 2**iii). In particular, the c.134-96T-allele was overrepresented in the HIV-1-infected children compared to the exposed-uninfected children (58% *vs.* 42%; *P* = 0.001, *P*_Bonf_ = 0.016). The overrepresentation was primarily driven by the *in utero*-enriched (63% *vs.* 42%; *P* = 0.011, *P*_Bonf_ > 0.05) and mixed (59% *vs.* 42%; *P* = 0.004, *P*_Bonf_ > 0.05) infected groups (**Figure 2**iii). We combined the *in utero*-enriched and mixed transmission groups into a larger *in utero*-enriched group, excluding the 27 HIV-1 infected and 75 HIV-1 exposed uninfected breastfed individuals, and still observed overrepresentation of the minor allele in the HIV-1 infected children (60% *vs.* 43%; *P* = 0.002, *P*_Bonf_ = 0.048) (data not shown).

The association between *FCGR2C* c.134-96C>T variant and HIV acquisition was assessed in a univariate model and a multivariate model that controlled for birthweight and CNR1 copy number, which were statistically significant at univariate analysis (**Table 4**). In a dominant model, the c.134-96C>T minor allele was associated with increased odds of perinatal HIV-1 transmission at univariate (OR = 1.89; 95% CI 1.31-2.73; *P* = 0.001, *P*_Bonf_ = 0.016) and multivariate analysis (AOR = 1.89; 95% CI 1.25-2.87; *P* = 0.003, *P*_Bonf_ = 0.048). The association was specific to the *in utero*-enriched (OR = 2.34; 95% CI 1.24-4.42; *P* = 0.009, *P*_Bonf_ > 0.05) and the mixed transmission groups (OR = 1.94; 95% CI 1.24-3.06; *P* = 0.004, *P*_Bonf_ > 0.05) but not the *in utero* (OR = 1.24; 95% CI 0.49-3.12; *P* = 0.653) and intrapartum groups (OR = 1.29; 95% CI 0.62-2.69; *P* = 0.500). Statistical significance was retained in both *in utero*-enriched (AOR = 2.49; 95% CI 1.31-4.76; *P* = 0.006, *P*_Bonf_ > 0.05) and mixed transmission groups (AOR = 2.06; 95% CI 1.28-3.30; *P* = 0.003, *P*_Bonf_ = 0.048) after adjusting for birthweight.

**Table 4:** Univariate and multivariate analysis of the effect of *FCGR2C* c.134-96C>T on perinatal acquisition of HIV-1.

Genotype	Univariate		Multivariate	
	OR (95% CI)	*P* value	Adjusted OR (95% CI)	*P* value
Total infected*				
CC	Ref		Ref	
CT/TT	1.89 (1.31-2.73)	**0.001 (*P*_Bonf_ = 0.016)**	1.89 (1.25-2.87)	**0.003 (*P*_Bonf_ = 0.048)**
*In utero-enriched* ^#^				
CC	Ref		Ref	
CT/TT	2.34 (1.24-4.42)	**0.009** (*P*_Bonf_ = 0.144)	2.49 (1.31-4.76)	**0.006** (*P*_Bonf_ = 0.064)
*Mixed* ^#^				
CC	Ref		Ref	
CT/TT	1.94 (1.24-3.06)	**0.004** (*P*_Bonf_ = 0.064)	2.06 (1.28-3.30)	**0.003 (*P*_Bonf_ = 0.048)**
*In utero*				
CC	Ref			
CT/TT	1.24 (0.49-3.12)	0.653		
*Intrapartum*				
CC	Ref			
CT/TT	1.29 (0.62-2.69)	0.500		

OR, Odds Ratio; CI, Confidence Interval; P_Bonf_, Bonferroni corrected P value.

Bold indicates statistical significance of P < 0.05.

*adjusted for birthweight and CNR1 copy number.

^#^adjusted for birthweight.

### The *FCGR2C* c.134-96C>T and c.169T>C Are in High Linkage Disequilibrium

**Figure 3:**
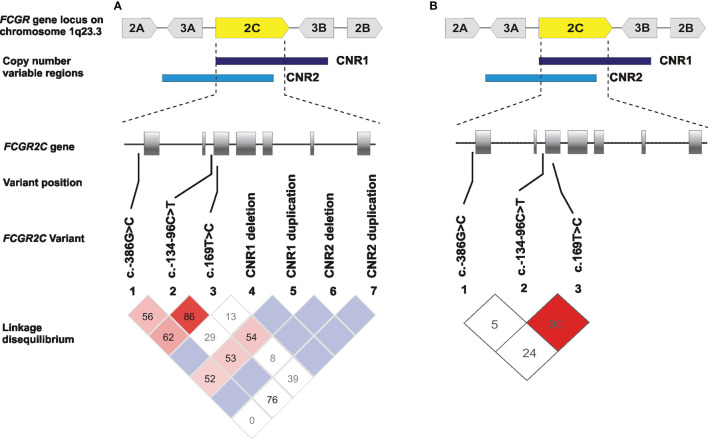
Linkage disequilibrium of the single nucleotide polymorphisms (SNPs) and copy number regions (CNRs) in *FCGR2C* gene in South African children born to women living with HIV-1. **(A)** LD plots for SNPs without considering CNV and **(B)** when only those with two gene copies were included. Values reflect D′ measures of LD and color in the squares given by standard D′/LOD (log of the odds of there being LD between two loci) color scheme, with bright red color used to display very strong LD.

The observed genotype frequencies for *FCGR2C* c.–386G>C, c.134-96C>T, c.169T>C were in Hardy-Weinberg equilibrium (*P* > 0.05). We also observed that 71% (39/55) of children carrying a c.169C were heterozygous for the *FCGR2B/C* promoter variant (data not shown). We analyzed linkage disequilibrium between the *FCGR2C* variants and CNRs, with and without considering the CNV. It was important to determine whether the observed variants associated with HIV-1 acquisition act independently or linkage disequilibrium plays a part. Our result demonstrated high linkage disequilibrium between c.134-96C>T and c.169T>C both without considering CNV (D′ = 0.867; r^2^ = 0.319) and when only those with two gene copies were included (D′= 0.908 and r^2^ = 0.213) (**Figure 3**). Both c.134-96C>T and c.169T>C independently associated with increased odds of HIV-1 acquisition, but in multivariate analysis, c.134-96C>T retained significance (AOR = 1.91; 95% CI 1.23-2.96; *P* = 0.004, *P*_Bonf_ > 0.05) while c.169T>C did not (AOR = 1.14; 95% CI 0.70-1.86; *P* = 0.590).

### Effect of *FCGR2C* c.134-96C>T and Maternal Viral Load on Perinatal Acquisition of HIV-1

Maternal viral load is a key determinant of MTCT of HIV-1 infection. For the NEVEREST cohorts, the maternal viral load was not recorded. However, in the birth cohort with the defined modes of transmission, information on maternal HIV-1 viral load was available for 279 mothers of whom 70 transmitted HIV to their infants and 209 did not. The mean HIV-1 viral load was significantly higher in transmitting mothers than non-transmitting mothers (4.53 vs. 3.95 log_10_ copies/ml; *P* < 0.0001). The maternal *FCGR2C* c.134-96C>T variant independently associated with HIV-1 transmission (OR = 1.98; 95% CI 1.16-3.37; *P* = 0.012, *P*_Bonf_ = 0.192) and when controlled for maternal viral load and birthweight (AOR = 2.03; 95% CI 1.14-3.62; *P* = 0.016, *P*_Bonf_ = 0.256). In this small subset, the infant *FCGR2C* c.134-96C>T variant was independently associated with HIV-1 acquisition (OR = 1.92; 95% CI 1.14-3.23; *P* = 0.014, *P*_Bonf_ = 0.224). The significant association remained when adjusted for maternal viral load (AOR = 2.10; 95% CI 1.13-3.87; *P* = 0.018, *P*_Bonf_ = 0.288), specifically in the *in utero*-enriched transmission group (AOR = 2.67; 95% CI 1.33-5.37; *P* = 0.006, *P*_Bonf_ = 0.064) (**Table 5**).

**Table 5:** Effect of *FCGR2C* c.134-96C>T on perinatal acquisition of HIV-1 in study cohort with information on maternal viral load.

Genotype	Univariate		Multivariate	
	OR (95% CI)	*P* value	Adjusted OR (95% CI)	*P* value
Total infected*				
CC	Ref		Ref	
CT/TT	1.92 (1.14-3.23)	**0.014** (*P*_Bonf_ = 0.224)	2.10 (1.13-3.87)	**0.018** (*P*_Bonf_ = 0.288)
*In utero-enriched* ^#^				
CC	Ref		Ref	
CT/TT	2.34 (1.24-4.42)	**0.009** (*P*_Bonf_ = 0.144)	2.67 (1.33-5.37)	**0.006** (*P*_Bonf_ = 0.064)

OR, Odds Ratio; CI, Confidence Interval; P_Bonf_, Bonferroni corrected P value.

Bold indicates statistical significance of P < 0.05.

*adjusted for maternal viral load, birthweight and CNR1 copy number.

^#^adjusted for maternal viral load and birthweight.

### Mother-Child *FCGR2C* c.134-96C>T Genetic Similarity and HIV-1 Acquisition

We next evaluated mother-child *FCGR2C* c.134-96C>T genotype concordance and association with HIV-1 acquisition. Concordance was defined as mother and infant each bearing at least one T allele (mother-child: CT/TT-CT/TT) or both were homozygous for the C allele (CC). Discordance was defined as one individual within the dyad possessing a CC genotype and the other bearing a T allele (CT/TT). Possession of a T allele in both mother and infant was overrepresented in the HIV-1-infected children compared to the exposed-uninfected children (45% *vs.* 28%; *P* = 0.012, *P*_Bonf_ = 0.192). Independently, MTCT was more likely among the mother-child concordant CT/TT group compared to the concordant CC group (OR = 2.58; 95% CI 1.36-4.88; *P* = 0.004, *P*_Bonf_ = 0.064) and retained significance after adjusting for maternal viral load, birthweight and infant CNR1 copy number (AOR = 2.87; 95% CI 1.36-6.06; *P* = 0.006, *P*_Bonf_ = 0.096) (**Table 6**).

**Table 6:** Mother-child *FCGR2C* c.134-96C>T genetic similarity and HIV-1 acquisition^a^.

Genotype (mother-child pair)	HIV-1-exposed uninfected n = 222	HIV-1 infected n = 76	Univariate		Multivariate*	
			OR (95% CI)	*P* value	OR (95% CI)	*P* value
Concordant CC	94 (42)	20 (26)	Ref		Ref	
Concordant CT/TT	62 (28)	34 (45)	2.58 (1.36-4.88)	**0.004** (*P*_Bonf_ = 0.064)	2.87 (1.36-6.06)	**0.006** (*P*_Bonf_ = 0.096)
Discordant	66 (30)	22 (29)	1.57 (0.79-3.10)	0.197	1.50 (0.68-3.29)	0.308

OR, Odds Ratio; CI, Confidence Interval; P_Bonf_, Bonferroni corrected P value.

Bold indicates statistical significance of P < 0.05.

*adjusted for maternal viral load, birthweight and CNR1 copy number.

a “Concordant” denotes mother-child pairs with same FCGR2C c.134-96C>T genotype. “Discordant” denotes mother-child pairs with different genotypes.

## Discussion

Previous studies have reported both functional and clinical relevance of *FCGR2C* genetic variability, including single nucleotide polymorphisms and copy number variations. Expression of the membrane-bound FcγRIIc protein in individuals bearing the *FCG2C* c.169T>C minor allele (*FCGR2C*-ORF) has been associated with susceptibility to idiopathic thrombocytopenic purpura (15), Kawasaki disease (32), systemic lupus erythematosus and increased antibody responses to vaccinations (11). Furthermore, the *FCGR2C* c.134-96C>T tag variant associated with reduced risk of HIV-1 infection in the Thai phase 3 RV144 HIV-1 vaccine trial (13) and increased risk of HIV-1 disease progression in black South Africans (18). In addition, increased risk of HIV-1 acquisition in the HVTN 505 vaccine trial was associated with two variants within the Thai *FCGR2C* haplotype but not the c.134-96C>T tag variant (17).

In this study, we report further associations between *FCGR2C* variants and perinatal HIV-1 acquisition in South African children. Deletion of CNRI was significantly protective of perinatal HIV-1 acquisition compared to two gene copies, but the significance was not retained after Bonferroni correction. However, the observed association between CNR1 and perinatal HIV-1 acquisition might not be due to *FCGR2C* copy number variability because *FCGR3B* and *HSPA7* genes are also deleted with CNR1. The associations appear to be unrelated to surface expression of membrane-bound FcγRIIc. While some children carried the c.169C open reading frame allele, the co-occurrence of the splice-site variant c.798+1A allele predicted the absence of FcγRIIc expression in the majority of children. The latter allele gives rise to alternative mRNA splicing and a premature stop codon with associated loss of surface expression (6, 32). Only four children carried both the c.169C open reading frame allele and the splice-site variant c.798+1G allele required for functional expression of FcγRIIc. This finding correlates with previous studies that suggested rare to absent expression of FcγRIIc in the black African population (12, 32) and raises questions around the functional relevance of the c.169T>C variant.

The *FCGR2C* c.134-96T-minor allele was associated with increased odds of perinatally acquired HIV-1 acquisition in South African children. Specifically, a significant association was observed in the *in utero*-enriched and mixed transmission groups but not in the *in utero* and intrapartum transmission groups. The *in utero*-enriched and mixed transmission groups are very similar in terms of drug treatment, as all were exposed to nevirapine for prevention of MTCT. Due to the nevirapine, fewer infants in the mixed group would have had intrapartum transmission and therefore were likely *in-utero* enriched. After adjusting for multiple comparisons using Bonferroni correction, the overall c.134-96C>T association retained significance, primarily driven by the mixed transmission group. The non-significance in the *in utero*-enriched group may potentially be due to small sample size. We postulate that the role of c.134-96C>T in HIV-1 acquisition is more pronounced during the course of pregnancy and at the maternal-fetal interface (3). In a subset of our study cohort, the observed association with *FCGR2C* c.134-96C>T variant remained when adjusted for maternal viral load, a key determinant of MTCT of HIV-1 infection. In both mother and child, the *FCGR2C* c.134-96C>T variant was associated with HIV-1 transmission and acquisition, respectively. Concordance in mother-child possession of the c.134-96T-allele further associated with a greater risk of MTCT compared to homozygosity for the C-allele in both mother and infant.

Assessing the role of the *FCGR2C* c.134-96C>T tag variant in both HIV-1 acquisition and disease progression has produced contrasting results of both protective and deleterious effects. The Thai phase 3 RV144 HIV vaccine trial provided the first clinical evidence of vaccine-induced protection, where the *FCGR2C* c.134-96C>T tag variant reduced the risk of HIV acquisition (13). Subsequently, two variants within the Thai *FCGR2C* haplotype were associated with increased risk of HIV-1 acquisition in vaccine recipients in the HVTN 505 trial but the c.134-96C>T tag variant was not. The population in this trial was predominantly Caucasian men who have sex with men (17). The *FCGR2C* c.134-96C>T tag variant was also associated with HIV-1 disease progression in South African adults (18). In this mother-child transmission model study, we establish its role in increased predisposition to HIV-1 acquisition. Whether the contrasting associations bear any functional significance is currently not clear and the modulating risk factors may not necessarily overlap (18). The differing results may be attributable to a variety of factors, including genetic differences between populations. It has been shown that two of the three variants within the Thai *FCGR2C* haplotype are absent in the African population, including black South Africans (12).

These findings may be of consequence to efforts aimed at elucidating the different outcomes of the two very similar HIV-1 vaccine efficacy trials, RV144 in Thailand and HVTN702 in South Africa. In addition to population genetic differences, the vaccine regimen evaluated in the RV144 and HVTN702 vaccine efficacy trials were also different. The RV144 vaccine candidate was specific for HIV-1 clades B and E with alum as adjuvant. This vaccine candidate elicited robust cross-clade immune responses in South Africans (33). However, in subsequent clinical trials the RV144 vaccine regimen was modified to increase immunogenicity and improve the duration of vaccine-induced immune responses (34). The vaccine regimen was HIV-1 clade C-specific, the predominant clade in South Africa, and was adjuvanted with MF59 (34, 35). The adapted ALVAC-HIV and Bivalent Subtype C gp120–MF59 vaccine regimen evaluated in HVTN 702 trial showed no efficacy among South African adults, despite previous evidence of immunogenicity (19). The differential vaccine efficacy between the RV144 and HVTN 702 trials might be due to differences in some components of the vaccine regimen and host genetics. In vaccine design, it is important to consider host genetic variation that can modulate vaccine efficacy (36). We previously established that black South Africans do not possess the complete Thai *FCGR2C* haplotype and are only polymorphic for c.134-96C>T (rs114945036) (12).

The functional mechanisms underlying the association of the variants within the Thai *FCGR2C* haplotype with HIV-1 acquisition, disease progression and vaccine efficacy is largely undefined. It raises many biological questions as to how expression of the membrane-bound FcγRIIc protein or the variants could modulate HIV-1 infection. The proposed impact of the *FCGR2C* variant haplotypes on immunity against HIV is unknown, one can only speculate. It is increasingly evident that it does not involve expression of the surface FcγRIIc receptor, since a limited number of individuals in the available genetic association studies bear the minor alleles that predict expression of the surface molecule. It is unknown whether a truncated soluble form of the FcγRIIc protein is produced in individuals with the premature stop codon or alternative splicing variants that prevent surface expression of FcγRIIc. Results from a study suggest that variants within the Thai *FCGR2C* haplotype either directly associate with the expression of *FCGR2C* in human B cells or in correlation with other causal variants that affect expression levels (36). The correlation with *FCGR2A* was observed across different populations and was specific to the c.134-96C>T variant (36, 37). We have proposed that the variants modulate transcription factor binding that may alter mRNA expression (18). This may in turn affect processes regulated by mRNA from the *FCGR2C* gene.

In conclusion, the *FCGR2C* variant c.134-96T-allele, which was associated with protection in the Thai RV144 trial, was associated with increased odds of perinatal HIV-1 acquisition in black South African children. This adds to other deleterious associations found for *FCGR2C* variants in the context of HIV-1 (17, 18) and warrants investigation of these variants in South African adults actively immunized in the HVTN 702 trial (19) and individuals passively immunized with the broadly neutralizing VRC01 antibody in the Antibody Mediated Prevention (AMP) trials (38). Collectively, these intriguing results highlight the need for further mechanistic studies to establish the functional relevance of *FCGR2C* variation in different populations and more broadly in contexts of vaccination and disease.

## Data Availability Statement

The raw data supporting the conclusions of this article will be made available by the authors, without undue reservation.

## Ethics Statement

Ethics approval for the study was obtained from the University of the Witwatersrand Human Research Ethics Committee (Reference numbers: M170585; M180575). Written informed consent to participate in this study was provided by the participants’ legal guardian/next of kin.

## Author Contributions

RS, GG, and LK recruited study participants and acquired clinical data. MG extracted DNA from blood samples. JE and MP genotyped individuals. JE managed and analyzed the data. JE and RL wrote the first draft of the manuscript. CT and RL designed the study and supervised the research. All co-authors critically revised the manuscript for intellectual content. All authors contributed to the article and approved the submitted version.

## Funding

This study is supported in part by grants from the National Institutes of Child Health and Human Development (HD 42402, HD 47177, HD 57784, HD 073977 and HD 073952), Secure the Future Foundation, the South African Medical Research Council, and the South African Research Chairs Initiative of the Department of Science and Innovation and National Research Foundation (84177).

## Conflict of Interest

The authors declare that the research was conducted in the absence of any commercial or financial relationships that could be construed as a potential conflict of interest.

## Publisher’s Note

All claims expressed in this article are solely those of the authors and do not necessarily represent those of their affiliated organizations, or those of the publisher, the editors and the reviewers. Any product that may be evaluated in this article, or claim that may be made by its manufacturer, is not guaranteed or endorsed by the publisher.
